# Patient perspectives on self-monitoring of blood glucose: perceived recommendations, behaviors and barriers in a clinic sample of adults with type 2 diabetes

**DOI:** 10.1186/s40200-015-0172-z

**Published:** 2015-05-19

**Authors:** Jennifer E. F. Ward, Barbara A. Stetson, Sri Prakash L. Mokshagundam

**Affiliations:** Department of Psychological and Brain Sciences, 317 Life Sciences Building, University of Louisville, Louisville, KY 40292 USA; Department of Medicine, University of Louisville, Louisville, KY 40292 USA

**Keywords:** SMBG, Blood glucose monitoring, Self-management, Type 2 diabetes, Barriers

## Abstract

**Background:**

Patient-centered perspectives on self-monitoring of blood glucose (SBMG) were assessed in adults with type 2 diabetes using a self-regulation conceptual framework.

**Methods:**

Participants (*N* = 589; 53 % female) were adults with type 2 diabetes who were recruited during routine appointments at a diabetes outpatient clinic in the Southeastern/lower Midwestern region of the United States.

**Results:**

Participant’s had varying perceptions regarding provider recommendations for SMBG (responder *n* = 380). Personal blood glucose testing patterns were also varied and reports frequently omitted (responder *n* = 296). Respondent’s most frequent personal pattern was to test “occasionally, as needed,” which did not differ by insulin use status, gender or age. In those *not* prescribed insulin, HbA1c reflected better control in those testing at least once per week (*p* = .040) or with a blood glucose goal (p = .018). 30.9 % endorsed at least monthly perceived encounters with SMBG barriers, with higher reports by women (*p* = .005) and younger (*p* = .006) participants. Poorer glycemic control was observed for participants with more frequently reported scheduling (*p* = .025, .041) and discouragement (*p* = .003) barriers.

**Conclusions:**

Findings suggest that many may experience difficulty integrating SMBG into their lives and are unsure of recommendations and appropriate function. Research is needed to promote best practice recommendations for SMBG.

## Background

Diabetes Mellitus (diabetes) is a rapidly increasing public health crisis, impacting over 300 million people globally [1]. When glucose is poorly controlled, risks of comorbidies, health complications and mortality are increased. In the face of the global epidemic, an understanding of approaches to stemming the tide of diabetes-related morbidity and mortality is critical [1]. Several large, prospective trials have revealed that many micro and macrovascular complications can be avoided or prevented with strict management of blood glucose control [2]. Studies suggest that much of the effective management of diabetes comes through intensive lifestyle change and regular monitoring of health behaviors and physical markers, such as weight and blood glucose levels, in the context of competing life priorities [3]. While research highlights the clear benefits of lifestyle change and the feasibility of achieving adherence to the demanding diabetes regimen, previous studies have been based on carefully selected participants. Translation of the behavioral management approaches used in clinical trials to real-world clinical settings has proved challenging to date [4] and is an important direction for research as interventions to assist individuals with diabetes in becoming engaged in the self-management of their diabetes can greatly help reduce risk of complications [5].

Self-monitoring of blood glucose levels (SMBG) is a key self-management component of the U.S. National Standards for Diabetes Self-Management and Education Practice Guidelines, which emphasize the critical role of behavioral goal-setting and decision-making [5, 6]. In recent years, there has been considerable debate about the effectiveness of the use of SMBG in the type 2 diabetes or non-insulin prescribed population [7]. Current standards of diabetes care and education emphasize the critical perspective of the person who is living with diabetes and advocate a patient-centered approach to care [2, 8]. While the resultant increase in research has called much-needed attention to the patient experience of SMBG and to SMBG behaviors, much of this research has not considered patient-centered perspectives or drawn on the decades of behavioral science literature on goal-setting, self-regulation, social ecological frameworks and problem solving [9]. Behavioral and psychosocial issues are much less likely to be addressed relative to routine clinical care and prevention and management of physical complications [10] and behavioral research in real world clinical settings lags behind recommendations. This discrepancy highlights the critical need for assessment of patient perspectives and SMBG related outcomes, using a patient-centered approach.

In order to address the limited consideration of patient perspectives on SMBG in clinical settings and studies, we propose that SMBG perceptions and behaviors be considered from a patient-centered perspective and a self-regulatory framework [11, 12]. Many factors related to patient characteristics and perspectives can be seen as contributing to the process of self-regulation such as knowledge, cultural background and beliefs, goal setting, emotional coping skill, and competing life priorities [13]. Recent studies have found that adults with type 2 diabetes who displayed stronger cognitive abilities, such as planning and problem solving, were more likely to engage in recommended self-management strategies than those with less well-developed skills [14] and that problem solving skills predicted better control of hyper and hypoglycemic episodes [15].

In addition to the need for theoretically based data on the perceptions and use of SMBG, data are needed to understand the actual SBMG practices of real-world clinical patients beyond the highly select participants in clinical trials. Surprisingly, little is known about patient perspectives on monitoring and their SMBG practices in clinic settings. Also, the limited translation of behavioral diabetes trials has constrained the integration of behavioral and social ecological assessments into clinical practice. By drawing from patient-centered research, behavioral and translational science may in turn, make a significant contribution to interventions that address the personal practices, barriers and patient outcomes most related to adherence to SMBG and move behavioral diabetes research toward interventions and practices that can increase self-management success.

## Study aims

The present study aims to increase understanding of patient perspectives on self-monitoring of blood glucose recommendations and their personal SMBG practices and experience of SMBG barriers within a self-regulation framework. Specifically, patient perceptions of provider recommendations and patient-reported SMBG behavioral practices and barriers are investigated in a diabetes clinic; associations between these factors and individual and illness characteristics are also examined.

## Method

### Design

The study used a cross-sectional design. Information pertaining to self-care behavior and health perceptions was obtained from a validated, self-report questionnaire. Type 2 diabetes status and insulin use/non-use and physical parameter data were obtained from medical chart review by trained clinic staff.

### Recruitment

The study was conducted at a hospital-based outpatient diabetes clinic in Southern Indiana in the Southeastern/lower Midwestern region of the United States. The clinic offers a variety of treatment, education, nutrition and exercise programs for patients with diabetes or endocrine disorders and their families.

Participants (*N* = 589, 53 % female) were recruited by clinic staff during routine outpatient clinic appointments at the time of check-in. Inclusion criteria were type 2 diabetes, 21 years though 90 years of age and able to read and write English. Exclusion criteria were type 1 diabetes, pregnancy, and cognitive impairment or severe mental illness precluding questionnaire completion. The study was approved by the University of Louisville Institutional Review Board. Informed consent was collected from all participants.

### Measures

The following were obtained from clinical medical chart data corresponding to the recruitment appointment: Height, weight and Hemoglobin A1c (HbA1c). HbA1c is an index of blood glucose control over the previous weeks to months; higher levels are a marker for poorly controlled diabetes and are associated with increased morbidity [16].

The Personal Diabetes Questionnaire (PDQ), a brief, clinically-focused, structured self-report measure of patient centered diabetes-related self-management behaviors, perceptions, and barriers [17] was completed by participants while waiting for clinic appointments or at home and returned by mail using pre-stamped envelopes. The PDQ scoring scheme is based on the behavioral domains of diabetes care, with subscales representing each self-care behavior. Select items and subscales were used to assess a number of self-regulatory factors including frequency of engaging in SMBG, knowledge (perceptions of provider recommendations), goal setting, barriers to SMBG (e.g. being too busy, feeling discouraged, dislike needles), and perceptions of glucose control.

### Statistical approach

All analyses were conducted using SPSS version 20.0 [18]. Participants were grouped dichotomously to reflect responses on items related to SMBG barriers and frequency (based on modal SMBG responses), insulin use status (whether or not they are prescribed injectable, subcutaneous insulin), gender and age group in order to statistically examine these variables as they relate to one another. Age was divided at 60 years due to sample distribution and research precedent [19]. For continuous variables (such as BMI and HbA1c), Two and Three-way ANOVA, *T*-test, and Chi-square were used. For ordinal and nominal data, Spearman’s Rho and Kruskal Wallis H-test analyses with Mann–Whitney U post hoc testing using the Holm corrections [20] were used. Holm corrections are appropriate for multiple comparisons of individual ordinal items that use Mann–Whitney U post hoc tests. The correction consists of a reduced p-value required for significance (p = 0.05/number of comparisons) with each required p-value increasing sequentially. For example, for an item with 6 ordinal response options, the results are first organized in order of significance. The finding with the lowest associated p-value is then compared with a required p-value for statistical significance of 0.05 divided by the total number of comparisons, or 6 (=0.00833). The second lowest p-value is then compared with 0.05 divided by the remaining number of comparisons, or 5 (=0.01). This process continues until the final finding (the sixth in this case) is compared with the uncorrected p-value (0.05).

## Results

### Sample characteristics

The sample included adults with type 2 diabetes, predominantly White American (96.1 %), with a mean age of 55.4 (*SD* = 13.04) years and 83.3 % with a high school degree. Having a subcutaneous insulin prescription (on insulin) was common (34.1 %)—even more so in those over 60 years of age [*X*^*2*^(2, 553) =7.324, *p* = .005] and female [*X*^*2*^(6, 553) =64.99, *p* < .001]. HbA1c (%) reflected moderate control [*M* = 8.614 (1.95)], consistent with a typical outpatient clinic sample (e.g. [21]) and was generally poorer [*F*(3, 584) =11.980, *p* = 0.001] in younger males [*M* = 8.65 (1.93)].

### Patient perceptions

#### Perceptions of provider recommendations

Participants were asked to respond to questions about provider recommendations and their actual typical frequency of engaging in SMBG; several participants left one or both of these items blank. Regarding perceptions of provider recommendations, 35.5 % of the sample left the item blank. Of those who did respond (*N* = 380), *n* = 14 (4 %) indicated that they (had) “not been told to test;” all but three of these individuals were not on insulin.

#### Perceptions of control

Control perceptions were generally supported by medical outcomes, with participants who rated their blood glucose control as ‘good’ to ‘excellent’ (26.3 %) tending to have lower HbA1c levels than those who indicated that they have ‘a few problems,’ ‘poor control,’ ‘very poor control,’ or did not know [*F*(1, 483) = 83.74, *p* < .001]. Many, in both insulin groups, reported having high blood sugar ‘nearly daily’ (37 % on insulin, 35.2 % other). Reports of low blood sugar were more common in those on insulin (33.2 % at least once per week) than those not on insulin [15.8 %; *F*(1, 430) =131.77, *p* < .001].

### Reported self-monitoring behaviors

A very large number of participants left an item reflecting SMBG frequency blank (50.3 %). Those not responding were excluded from subsequent SMBG behavior analyses but were examined for group features (i.e. differences in gender, age and insulin prescription status). The only significant difference was that the non-responders were more likely to be on insulin [*X*^*2*^(1, 552) =44.563, *p* < .001].

In those who responded (*n* = 296), SMBG frequency was reported as occurring at least one to two times weekly for 46.8 % (*n* = 203); the modal response was “occasionally, as needed.” Group differences were found when collapsing responses into dichotomous groups to compare those engaging in SMBG at least once per week and those engaging in SMBG less than once per week. Individuals prescribed insulin were more likely to report that they tested less than once per week (17.0 %) compared with those not using insulin [31.0 %; *F*(1, 552) =36.833, *p* < .001].

### Perceptions of barriers to self-monitoring

The majority of the sample (69.1 %) did not endorse barriers to SMBG that occurred more than once monthly. In those participants who reported that they engage in SMBG at least once per week (*n* = 136), higher overall SMBG barrier scores (reflecting perceptions of more frequent barriers) did not differ by insulin prescription but were associated with female gender [*F*(1, 136) = 8.292, *p* = .005] and younger age [*F*(1, 136) = 7.685, *p* = .006]. The endorsement of specific barriers was fairly consistent across age, gender and insulin groupings with barriers related to scheduling, such as being away from home or experiencing changed plans, as the most frequently and universally encountered. Higher scores on a schedule-related barrier subscale (α = .80), did not differ based on insulin prescription status or having a target range but were associated with younger age [*F*(1, 130) =10.712, *p* = .001] and female gender [*F*(1, 130) =5.502*, p* = .021], which is consistent with higher overall SMBG barriers reported in the young and female in this sample.

#### SMBG barriers and associations with testing frequency

Exploratory analyses examined whether perceptions of barriers differed by reported testing frequency and insulin use status. Based on observed differences in testing patterns, participants were divided into four groups: 1) those on insulin who reported that they engage in SMBG at least once per week (*n* = 29), 2) those on insulin engaging in SMBG less than once per week (*n* = 28), 3) those not on insulin engaging in SMBG at least once per week (*n* = 105) and 4) those not on insulin engaging in SMBG less than once per week (*n* = 90). No significant group differences were found in the reported degree of scheduling barriers to SMBG. More broadly, a subscale related to overall SMBG barriers differed by those on insulin only, *t*(254) =3.28, p = 0.002, with those engaging in SMBG less than once per week having significantly higher barrier scores [*M* = 2.40 (1.10)] indicating the perception of more frequent barriers to SMBG than those engaging in SMBG more frequently [*M* = 1.62 (0.63)]. This statistical difference remained significant after using a Holm correction for multiple comparisons. See Table 1 for a chart summarizing significant findings with Kruskal Wallis H-tests of ordinal data; Holm-corrected p-values are provided in the far right column.

**Table 1:** Significant Statistical findings for comparisons of Hemoglobin A1c (HbA1c) values by ordinal SMBG barrier frequencies ^ϯ^

Barrier to SMBG	Kruskal-Wallis test *X* ^2^	*P* value	Reported Barrier Level Group	*n*	HbA1c % Median, Mean (SE)	Man-Whitney U post hoc U=	*P* value	Holm- corrected significance value comparison
Away from Home	12.832	0.025	**4-6x/week**	**12**	**9.15, 9.63(0.44)**			
			1x/month or less	63	7.90, 7.88(0.26)	173.50	0.003*	0.008
			Never	278	7.90, 8.09(0.12)	851.00	0.004*	0.01
			2-3x/month	80	7.95, 8.27(0.19)	239.50	0.005*	0.0125
			1+ x/day	12	7.00, 7.42(0.50)	1854.00	0.007*	0.017
Discouraged	17.764	0.003	**Never**	**355**	**7.70, 7.95(0.10)**			
			1-3x/week	18	9.30, 9.47(0.52)	1854.00	0.003*	0.008
			2-3x/month	43	8.40, 8.57(0.24)	5925.00	0.017	0.01
Busy schedule^ϯ ϯ^	11.554	0.041	**Never**	**106**	**8.10, 8.19(0.18)**			
			4-6x/week	15	9.30, 9.63(0.41)	425.00	0.004*	0.008
			1-3x/week	17	9.10, 9.16(0.46)	619.00	0.039	0.01

*Note:* *indicates significance after compared with Holm corrected *p*-value

ϯ for a description of the statistical procedures used, please refer to the statistical approach section (above)

ϯ ϯ only participants prescribed insulin used in analyses

### Behaviors and perceptions associated with physiological outcomes

Reported frequency of SMBG was associated with better glycemic control in those not on insulin only, with those reporting that they engage in SMBG 1-2 times per week or more displaying lower HbA1c levels [*M* = 7.65(0.18)] relative to the other groups [*M* = 8.20(0.20); *F*(1, 226) = 4.287, *p* = .040]. Body Mass Index (computed from medical chart data) was significantly lower for those who engage in SMBG at least once per week in a group of older women [*F*(1, 33) =6.552, p = .016] but this difference was not observed in other gender and age groups.

#### Perceived goal setting

Those who endorsed the statement “I have a target range for my blood glucose” (58.6 %) had lower HbA1c [*M* = 7.96 (1.82)] levels than those who endorsed “no, I do not have a target range” [*M* = 8.34 (2.04)] or “I don’t know” [*M* = 8.38 (2.09); *F*(2, 552) =4.024, *p* = 0.18]. Of those who indicated that they had a target range for blood glucose, 43.15 % reported that they were asked by their healthcare provider to test their blood glucose levels at least once per week, compared with 54.35 % of those who did not have a target and 46.9 % who were not sure. Of those same responders, many in the “I have a target range” group left the item related to frequency of typical SMBG blank (60.17 %).

#### SMBG barriers and associations with glucose control

Specific barriers to SMBG related to cost, hating to stick oneself, family support and negative mood were not found to be associated with significant differences in metabolic control (HbA1c %) while specific barriers related to scheduling concerns and feeling discouraged were (Table 1). Specifically, being away from home several times per week was associated with poorer metabolic control than being away from home less frequently or at least once every day. In those on insulin, significantly better control was found in those *never* experiencing being “too busy” than in those who were “too busy” *several times per week*. Similarly, *never* “feeling discouraged” as a barrier to SMBG was associated with better metabolic control than experiencing the barrier *a few times per week*.

## Discussion

This study contributes to the behavioral diabetes literature in providing a theoretically based, patient-centered view of the SMBG experience in a clinical setting, which has been lacking in previous research [4]. Prior research points to the importance of considering patient perspectives in diabetes self-management practices [8, 22]. However, previous empirical studies have largely failed to examine patient perspectives of SMBG with patient samples from real-world clinical settings [23].

The findings of the present study reflect prevailing uncertainty—that many patients are unclear about SMBG recommendations, face practical barriers to testing and are unsure of how to integrate SMBG into their lives. The aim of the present study was to increase understanding of patient perspectives of, practices of, and barriers to SMBG within a self-regulation framework in a clinical setting. Following is a discussion of the findings from within this framework.

### Response rates

The finding that specific items related to SMBG recommendations were left blank by a number of participants (who did have a high rate of completion of items related to other aspects of diabetes self-care) is likely reflective of uncertainty about recommendations. Much can be learned from considering patterns of (non)responding, which may be viewed as analogous to considering rates of drop out from behavioral interventions rather than only focusing on those who successfully complete treatment. This pattern bears a resemblance to the lack of a consensus for specific SMBG recommendations for this population in the medical field [7].

### SMBG practices and associations

Approximately half of participants reported engaging in SMBG less than once or twice a week, if at all. Reported SMBG frequency was variable and similar for all participants, regardless of insulin prescription status. Further, more frequent engagement in SMBG was only associated with better metabolic control in those not prescribed insulin. There may be number of reasons for these findings. For one, those prescribed insulin tended to have poorer glucose control overall, leading to more varied HbA1c levels throughout group, which may have manifested in statistical concerns.

Though the perceived intended purpose of SMBG testing was not examined explicitly, control was better in those who indicated that they had a target for glucose control; using SMBG to achieve glucose targets appears to be critical to its effectiveness as a self-management tool. The findings point to the importance of using SMBG as part of a process of goal-directed, self-regulatory behavior.

### Demographic factors

Findings implicated gender and age as factors in the frequency of encountering difficult barriers to SMBG; this may reflect differences in perceptions and emotional responses to SMBG. For example, women reported a greater frequency of encountering barriers despite no indication of differences in SMBG frequency. Also, older adults reported fewer barriers than young adults, suggesting that characteristics common to older age, such a less chaotic lifestyle or different health expectations, allows for easier integration of SMBG into daily schedules. Due to study limitations, the relationships between gender and age and SMBG barriers cannot be adequately described. However, our results suggest that individual differences, such as gender, should be considered as a targeted outcome of interest for research or a consideration in clinical intervention.

### SMBG Barriers

Regardless of age, gender or insulin use status, SMBG barriers related to scheduling were the most commonly encountered and barriers related to cost and discomfort were among the lowest. Being away from home had the strongest relationship to metabolic control of the barriers examined; participants who were away from home several days in a week had poorer control than those away from home less frequently and, interestingly, those who were away from home every day had relatively good control. For those prescribed insulin, having a busy schedule was also related to poorer control, though this was not the case for those not prescribed insulin. Additionally, reports of practical and emotional barriers did not directly relate to poorer control. These findings highlight the complex experience of engaging in SMBG and barriers to SMBG. Individual, patient factors may be extremely important in the experience of SMBG.

To address barriers to SMBG, ideal treatment formats may differ depending on individual factors. A one-on-one treatment context can be useful for patients presenting with many specific concerns. Alternatively, patients can be organized into group interventions that can be tailored to address similar needs [4]. When a patient is presenting with difficulties that are mostly behavioral and related to adherence, and are not reporting a significant amount of emotional distress, a more behavioral approach can be used. See a conceptual overview and detailed description of a behavioral intervention for diabetes management in a helpful review by Peyrot and Rubin [9].

### Limitations

A significant challenge in the present study relates to the use of a self-report SMBG measure developed for clinical assessment. While useful for obtaining descriptive self-care information to inform treatment decisions, many items reflect ranked response options, yielding ordinal data, which necessitated the use of nonparametric analyses and a loss of statistical flexibility and power. It is recommended that future studies consider the inclusion of additional continuous variables that may allow for more detailed analyses related to SMBG testing and barrier frequency.

## Conclusions

Despite the above limitations, the present findings contribute to the literature by highlighting the patient experience of SMBG in individuals with Type 2 diabetes in a clinical setting from a theoretical perspective. Specifically, the findings point to frequent barriers experienced by patients, and the prevalent uncertainty surrounding recommendations. The finding that many patients face uncertainty suggests a great need for future research addressing the translation of conceptually grounded, best practice recommendations for SMBG into practice and behavioral intervention in clinical settings. An interdisciplinary care team and integration of behavioral research can contribute greatly to this endeavor by examining SMBG in clinical settings in a systematic way and developing evidence-based recommendations and strategies for addressing uncertainty and barriers to SMBG in these settings. Recommendations and considerations for future research and clinical translation are discussed in more detail below.

### Research recommendations

#### Research grounded in a conceptual model

Interdisciplinary diabetes care teams that include behavioral scientists or mental health practitioners can contribute greatly to our understanding of the patient experience of SMBG, and SMBG barriers, through expertise and application of any number of supported health behavior theories in existence. Application of these theories is needed to inform understanding of the psychological, social and behavioral processes that are involved in SMBG and can, in turn, inform translational medicine. The present study highlighted a complex relationship between individual characteristics, health behaviors, the experience of barriers and physiological outcomes. Conceptually grounded models can help to explain the behavioral, social and cognitive mechanisms that interplay and sustain these complex relationships.

#### Clear, operational definitions of optimal SMBG

Inconsistency in research definitions of SMBG has led to serious problems with inter-study comparison and applicability to clinical practice. In addition to the need to define SMBG frequency variables, the operational definition of the utility of SMBG should be considered (Is it being used to provide feedback to health care team? Is it being used to make behavioral changes? Is it lacking purpose?). This would allow for consideration of the underlying cognitive and coping process of SMBG in addition to behavioral tracking.

#### Clinical research

Examination of the efficacy of clinical intervention for diabetes-distress and adherence problems is needed. Specifically, it is as yet unknown whether some individuals will benefit more from a solely behavioral or solely support intervention rather than an intervention that integrates both foci [9].

#### Emphasize utility

While SMBG recommendations include timing and frequency, it is also beneficial to emphasize the utility of SMBG as a means of increasing personal involvement in diabetes care. Utility is gaining attention in recommendations [24]; SMBG may be discussed as both a momentary decision-making tool when away from the clinic working toward a personal target and as helpful in informing healthcare provider treatment choices in making clinical adjustments [25]. Indeed, our findings indicate that having a personal blood glucose target is associated with lower HbA1c, relative to not having a target or being uncertain of a personal target. However, there are very few research studies that emphasize the use of SMBG for decision-making, at present [26]. Further research should measure this component in a systematic way.

Barriers to SMBG can often be managed through informed clinical care and should be addressed before implementing any long-term SMBG regimen. There is burgeoning evidence that an integrated diabetes care team is improved by addressing behavioral and psychological needs.
